# Prevalence of non-alcoholic fatty liver disease and risk factors for advanced fibrosis and mortality in the United States

**DOI:** 10.1371/journal.pone.0173499

**Published:** 2017-03-27

**Authors:** Michael H. Le, Pardha Devaki, Nghiem B. Ha, Dae Won Jun, Helen S. Te, Ramsey C. Cheung, Mindie H. Nguyen

**Affiliations:** 1 Department of Health Research and Policy, Stanford University School of Medicine, Stanford, CA, United States of America; 2 Digestive Diseases Institute, Cleveland Clinic, Cleveland, OH, United States of America; 3 School of Medicine, University of California Davis, Sacramento, CA, United States of America; 4 Division of Gastroenterology and Hepatology, Stanford University Medical Center, Palo Alto, CA, United States of America; 5 Department of Gastroenterology, Hanyang University Medical Center, Seoul, Korea; 6 Section of Gastroenterology, Hepatology and Nutrition, Department of Medicine, The University of Chicago Medicine, Chicago, IL, United States of America; 7 Division of Gastroenterology and Hepatology, Veterans Affairs Palo Alto Health Care System, Palo Alto, CA, United States of America; Kaohsiung Medical University Chung Ho Memorial Hospital, TAIWAN

## Abstract

In the United States, non-alcoholic fatty liver disease (NAFLD) is the most common liver disease and associated with higher mortality according to data from earlier National Health and Nutrition Examination Survey (NHANES) 1988–1994. Our goal was to determine the NAFLD prevalence in the recent 1999–2012 NHANES, risk factors for advanced fibrosis (stage 3–4) and mortality. NAFLD was defined as having a United States Fatty Liver Index (USFLI) > 30 in the absence of heavy alcohol use and other known liver diseases. The probability of low/high risk of having advanced fibrosis was determined by the NAFLD Fibrosis Score (NFS). In total, 6000 persons were included; of which, 30.0% had NAFLD and 10.3% of these had advanced fibrosis. Five and eight-year overall mortality in NAFLD subjects with advanced fibrosis was significantly higher than subjects without NAFLD ((18% and 35% vs. 2.6% and 5.5%, respectively) but not NAFLD subjects without advanced fibrosis (1.1% and 2.8%, respectively). NAFLD with advanced fibrosis (but not those without) is an independent predictor for mortality on multivariate analysis (HR = 3.13, 95% CI 1.93–5.08, *p*<0.001). In conclusion, in this most recent NHANES, NAFLD prevalence remains at 30% with 10.3% of these having advanced fibrosis. NAFLD per se was not a risk factor for increased mortality, but NAFLD with advanced fibrosis was. Mexican American ethnicity was a significant risk factor for NAFLD but not for advanced fibrosis or increased mortality.

## Introduction

Nonalcoholic fatty liver disease (NAFLD) is becoming the most common cause of liver disease in Western countries and includes nonalcoholic steatohepatitis (NASH) which could progress to cirrhosis and is associated with liver cancer[1,2]. NAFLD is characterized by the presence of hepatic steatosis in the absence of excessive alcohol use or alternative cause of hepatic steatosis[2,3]. NAFLD is associated with diabetes, obesity, and hyperlipidemia and is considered to be the hepatic manifestation of metabolic syndrome[4–6]. Prevalence of NAFLD has been increasing in parallel with the prevalence of obesity, diabetes, and metabolic syndrome[7]. The prevalence of NAFLD in the United States (U.S.) has risen from 18% in 1988–1991 to 31% in 2011–2012[8]. Estimates of NAFLD prevalence for adults in Western countries is 20–30%, with much higher prevalence in adults with obesity (80–90%), diabetes (30–50%), and hyperlipidemia (90%)[9].

Hepatic steatosis is usually diagnosed by abdominal imaging, but several indices have also been developed to predict the presence of NAFLD using laboratory values and clinical data. The Fatty Liver Index (FLI) is such an index originally developed in Italy, consisting of triglycerides, body mass index (BMI), gamma-glutamyl transpeptidase (GGT), and waist circumference[10]. The FLI has since been validated in many other cohorts, including several population-based studies [11–16]. The United States Fatty Liver Index (USFLI) consists of age, ethnicity, GGT, waist circumference, fasting glucose, and fasting insulin was more recently developed. By including ethnicity, the USFLI has been shown to be more reliable than FLI in predicting NAFLD in the multi-ethnic U.S. population [8]. As such, the USFLI can be useful in identifying U.S. patients with NAFLD without liver biopsy or ultrasound.

Though liver biopsy remains the gold standard to diagnose NASH and staging of fibrosis, it is expensive, invasive and subject to sampling error, limiting its use in clinical practice[2]. Therefore, non-invasive assessments such as the NAFLD Fibrosis Score (NFS) was developed. An NFS cutoff of > 0.676 has been shown to have a positive predictive value of advanced fibrosis of 82–90%[17]. The NFS determination of advanced fibrosis has been validated in 13 studies that included more than 3000 patients[18], and it is currently the most accurate non-invasive test for predicting advanced fibrosis for NAFLD in comparison studies[19–20]. The NFS has also been endorsed by both the European Association for the Study of the Liver and the American Association for the Study of Liver Diseases to risk stratify the need for liver biopsy in patients with NAFLD[2,21]. As such, the NFS was chosen as the non-invasive assessment of choice for determining the presence of advanced fibrosis in this study.

Previous studies utilizing the NHANES database have examined mortality of patients recruited from the 1988–1994 cycle. The aims of current study are to determine t the prevalence of NAFLD and advanced fibrosis in NAFLD in a more recent US sample from the NHANES 1999–2012 cycle and to identify predictors for NAFLD, advanced fibrosis and mortality of NAFLD subjects. We use the USFLI to identify subjects with NAFLD and NFS to identify those with advanced fibrosis and to determine risk factors associated with advanced fibrosis in a large population-based multiethnic U.S. cohort.

## Patients and methods

The National Health and Nutrition Examination Survey (NHANES) is conducted in the U.S. by the National Center for Health Statistics (NCHS) of the Centers for Disease Control and Prevention (CDC)[22]. NHANES has been a continuous survey in 2-year cycles starting from 1999[23]. The survey consists of cross-sectional interview, examination, and laboratory data collected from a complex multistage, stratified, clustered probability sample representative of the civilian, non-institutionalized population with oversampling of certain subgroups during different time periods. The survey was approved by the institutional review board of the CDC, and all participants provided written informed consent to participate.

This study represents an analysis of the continuous NHANES data between 1999 and 2012. Participants were included if they were 18 years or older and attended a medical examination at a mobile center after an overnight fast. Of the 41,243 sampled persons aged 18 years and older, a total of 39,175 subjects attended an examination at a mobile examination center. Of these, 16,644 persons were examined in the morning after an overnight fast with fasting laboratory testing and met inclusion criteria. Participants were excluded if they tested positive for hepatitis B core antibody, hepatitis C antibody, hepatitis C virus RNA (n = 1311), if data was missing on hepatitis serology (n = 116) or on alcohol use (n = 3532), on GGT, waist circumference, serum insulin, or serum glucose (n = 233) and persons with significant alcohol intake (>2 drinks per day for men or >1 drink per day for women, n = 5452). In total, 6000 persons met all criteria and were included in the analysis for our study.

General demographic characteristics collected during the interview included age (years), sex, ethnicity (non-Hispanic White, Non-Hispanic Black, Mexican American, Other Hispanic, Other Race–other non-Hispanic race including non-Hispanic multiracial), country of birth, citizenship status, military status, income (<10,000, 10,000–44,999, 45,000–74,999, 75,000+ dollars), poverty income ratio (<1.0, ≥1.0), marital status (legally married, divorced/separated, never married, other–widowed/living with partner), exposure to smoking (former/current, never), and educational level(≤high school, >high school degree). BMI [weight (kg) / height (m^2^)], and waist circumference (cm) data were collected during the medical examination in the mobile examination center. Serum was tested for albumin (g/dL), alanine aminotransferase (ALT, U/L), aspartate (aminotransferase (AST, U/L), alkaline phosphatase [ALP, U/L), gamma-glutamyl transpeptidase (GGT, U/L), platelet count (1000 cells/µL), total bilirubin (mg/dL), creatinine (mg/dL), hemoglobin A_1C_ (HbA1c, %), fasting glucose (mg/dL), fasting insulin (uU/mL), total cholesterol (mg/dL), high density lipoprotein (HDL) cholesterol (mg/dL), low density lipoprotein (LDL) cholesterol (mg/dL), and triglycerides (mg/dL).

NHANES mortality data is available for the years 1999–2010. Of the 5,094 NHANES participants in the 1999–2010 cycles who met previous inclusion criteria, 5,086 participants had available data on mortality and were included in the mortality analysis (84.8% of the total study sample of 6000). Participants were passively followed through December 31, 2011, by linking continuous NHANES participants through the National Death Index by probabilistic record matching[24]. Mortality outcomes were based on the stated underlying or other cause of death on the death certificate, coded according to the *International Classification of Diseases*, Tenth Revision (ICD-10) for deaths occurring between 1999 and 2011. Outcomes for this analysis consisted of all-cause mortality and cause-specific mortality from diseases of heart (ICD-10 codes I00-I09, I11, I113, I20-I51), and malignant neoplasms (ICD-10 codes C00-C97). The 2011 Public-Use version of Linked Mortality Files were used for this analysis. Public use data files include participants aged 18 and older with a limited set of mortality variables in addition to the perturbation of data to reduce the risk of re-identification of NHANES participants[25].

Factors with potential influence on presence of liver fibrosis were included as covariates in the multivariate analyses: sex, ethnicity, education level and smoking status [17, 26].

The diagnosis of NAFLD was ascertained among participants aged 18 and above by using the United States Fatty Liver Index (USFLI). The USFLI was calculated as described [8]:

USFLI=e^(−0.8073*non−HispanicBlack+0.3458*MexicanAmerican+0.0093*Age+0.6151*log_e(GGT)+0.0249*WaistCircumference+1.1792*log_e〖(Insulin)+〗0.8242*log_e〖(Glucose)−14.7812〗)/((1+e^(−0.8073*non−HispanicBlack+0.3458*MexicanAmerican+0.0093*Age+0.6151*log_e(GGT)+0.0249*WaistCircumference+1.1792*log_e〖(Insulin)+〗0.8242*log_e〖(Glucose)−14.7812〗)))*100

where “non-Hispanic Black” and “Mexican American” have a value of 1 if the person is of that ethnicity and 0 if the person is not. The USFLI has been validated and shown to correlate well with the presence of NAFLD diagnosed through ultrasound (AUROC of 0.80; 95% CI = 0.77–0.83)[8]. Using the recommended values, a score of USFLI ≥30 was selected to rule in fatty liver.

### Definitions

Hypertension was defined as having systolic blood pressure ≥140 mmHg or diastolic blood pressure ≥90 mmHg. Hypercholesterolemia was defined as having LDL cholesterol ≥130 mg/dL while hyperlipidemia was diagnosed as having triglycerides ≥150 mg/dL. Metabolic syndrome was defined according to the National Cholesterol Education Program (NCEP) ATP-III Guidelines[27].

Diabetes mellitus was defined as physician diagnosed diabetes or fasting plasma glucose ≥126 mg/dL. Controlled diabetes was defined as the participant having diabetes with HbA1c <6.5% while uncontrolled diabetes was defined as the participant having diabetes with HbA1c ≥6.5%. Impaired fasting glucose was defined as having a fasting plasma glucose ≥110 mg/dL. The homeostatic model assessment of insulin resistance (HOMA-IR) was calculated using the standard equation[28].

Kidney failure, asthma, arthritis, ischemic heart disease, congestive heart failure, stroke, chronic obstructive pulmonary disease (COPD), and cancer were ascertained through physician diagnosis. COPD was defined as having either emphysema or chronic bronchitis.

Advanced fibrosis was defined as having an NFS >0.676. The NFS was calculated as described [17]:

$$\begin{array}{l} \;NFS=\\ -1.675+0.037*age(years)+0.094*BMI\left(\frac{kg}{m^{2}}\right)+1.13*\\ (impaired\;fasting\;glucose\;or\;diabetes)+0.99*\left(\frac{AST}{ALT}\right)-0.013*platelet\left(\frac{x109}{L}\right)-0.66*\\ albumin\left(\frac{g}{dL}\right) \end{array}$$

where impaired fasting glucose/diabetes has a value of 1 if the subjects have impaired fasting glucose or diabetes and a value of 0 if they do not.

### Statistical analysis

Descriptive statistics were reported as proportion (%) for categorical variables, and mean ± standard deviation for continuous variables. Categorical variables were evaluated using the chi-square (*X*^2^) test. Normally distributed continuous variables were evaluated using the student *t*-test. Continuous variables that were not normally distributed were evaluated using nonparametric methods. Independent predictors of NAFLD diagnosis based on the USFLI or advanced fibrosis based on the NFS were evaluated with univariate and multivariate logistic regression inclusive of age, sex, ethnicity, education level, smoking status, BMI, diabetes status, and metabolic syndrome. The validation of the Cox proportional hazards assumption was performed through graphical comparison of the Kaplan-Meier survival curves with the Cox predicted curves for the same variable. If the predicted or observed curves were close together, the proportional-hazards assumption was not violated[29]. Statistical significance was defined with a two-tailed *p*-value ≤ 0.05. All statistical analysis was performed using Stata 11.2 (Stata Corporation, College Station, TX, USA), which allows appropriate use of the stratified sampling design employed by NHANES to project the data to the United States population[23, 30–31].

Weighted analyses were carried out using survey weights created in NHANES. These weights are used to account for the complex survey design, survey non-response, post-stratification, and oversampling. By weighting the sample, then that sample becomes representative of the U.S. non-institutionalized population[32]. For survival analysis, weighted analysis for total U.S. population estimates were performed.

## Results

### Demographic and laboratory characteristics

Demographic characteristics of subjects with and without NAFLD are summarized in Table 1, while clinical and laboratory characteristics are summarized in Table 2. Subjects with NAFLD were more likely to be male, older in age, Mexican American, born in the U.S., legally married and to have previously served in the U.S. military, to have lower income, previous smoking exposure, and a lower education level (Table 1). Subjects with NAFLD were also more likely to have hypertension, hypercholesterolemia, hyperlipidemia, metabolic syndrome, diabetes, asthma, arthritis, ischemic heart disease, congestive heart failure, stroke, COPD, and cancer. Subjects with NAFLD were also more likely to have higher BMI, waist circumference, NFS, ALT, AST, ALP, GGT, platelet, creatinine, fasting glucose, fasting insulin, HOMA-IR, and triglycerides (Table 2).

**Table 1:** Demographic characteristics of participants by NAFLD status in the U.S. National Health and Nutrition Examination Survey, 1999–2012.

Variable	NAFLD (n = 1,936)	No NAFLD (n = 4,064)	*p-*value
Age (years)	53.2 ± 16.6	47.3 ± 16.9	<0.001
Male	59.2%	45.5%	<0.001
Ethnicity			<0.001
Non-Hispanic White	77.6%	73.6%	
Non-Hispanic Black	5.7%	11.6%	
Mexican American	9.0%	4.9%	
Other Hispanic	4.0%	4.0%	
Other race	3.7%	5.9%	
Foreign born	13.0%	16.0%	0.004
United States citizen	93.2%	91.7%	0.07
Served in the U.S. military	18.9%	11.2%	<0.001
Income			0.006
< $45,000	43.8%	36.5%	
$45,000–74,999	24.2%	25.2%	
≥ $75,000	32.0%	38.3%	
Poverty income ratio < 1.0	10.5%	10.2%	0.81
Marriage status			0.005
Legally married	67.5%	64.0%	
Divorced/separated	9.4%	9.1%	
Never married	10.7%	15.3%	
Other	12.5%	11.6%	
Smoking exposure			<0.001
Never	58.7%	65.6%	
Current/former	41.3%	34.4%	
Education level			<0.001
≤ High school	41.8%	35.2%	
> High school degree	58.2%	64.8%	

**Table 2:** Lab and clinical characteristics of participants by NAFLD status in the U.S. National Health and Nutrition Examination Survey, 1999–2012.

Variable	NAFLD (n = 1,936)	No NAFLD (n = 4,064)	*p-*value
Hypertension	20.8%	13.6%	<0.001
Hypercholesterolemia	35.9%	33.6%	0.006
Hyperlipidemia	50.9%	20.6%	<0.001
Metabolic syndrome	56.1%	14.1%	<0.001
Diabetes	22.5%	4.4%	<0.001
Controlled (HbA1c<6.5%)	9.7%	2.2%	<0.001
Uncontrolled (HbA1c≥6.5%)	12.8%	2.2%	
Kidney failure	2.1%	1.6%	0.17
Asthma	15.0%	11.8%	0.004
Arthritis	37.0%	22.0%	<0.001
Ischemic heart disease	11.7%	4.2%	<0.001
Congestive heart failure	4.2%	1.2%	<0.001
Stroke	3.8%	1.7%	<0.001
Chronic obstructive pulmonary disease	8.8%	4.8%	<0.001
Cancer	12.2%	8.8%	0.001
BMI (kg/m^2^)	33.8 ± 6.8	25.9 ± 4.5	<0.001
Waist circumference (cm)	112.7 ± 14.6	90.9 ± 11.7	<0.001
NFS	-1.28 ± 1.58	-2.34 ± 1.33	<0.001
Low probability	44.0%	75.6%	<0.001
Indeterminate	45.7%	22.5%	
High probability	10.3%	1.9%	
Albumin (g/dL)	4.2 ± 0.3	4.3 ± 0.3	<0.001
Alanine aminotransferase (U/L)	31.6 ± 19.9	21.6 ± 10.2	<0.001
Aspartate aminotransferase (U/L)	27.0 ± 12.7	23.4 ± 8.9	<0.001
Alkaline phosphatase (U/L)	73.4 ± 22.2	65.4 ± 20.0	<0.001
Gamma-glutamyl transferase (U/L)	38.5 ± 39.0	19.6 ± 13.8	<0.001
Platelet (10^9^/L)	259.6 ± 68.3	251.4 ± 62.4	<0.001
Total bilirubin (mg/dL)	0.76 ± 0.31	0.78 ± 0.31	0.09
Creatinine (mg/dL)	0.92 ± 0.31	0.87 ± 0.36	<0.001
HbA1c	5.9 ± 1.1	5.4 ± 0.6	<0.001
Fasting glucose (mg/dL)	115.7 ± 35.7	97.1 ± 16.0	<0.001
Fasting insulin (uU/mL)	20.9 ± 16.7	7.8 ± 3.8	<0.001
HOMA-IR	6.0 ± 5.8	1.9 ± 1.0	<0.001
Total cholesterol (mg/dL)	199.8 ± 44.2	197.4 ± 39.3	0.17
HDL cholesterol (mg/dL)	45.4 ± 11.9	56.6 ± 15.1	<0.001
LDL cholesterol (mg/dL)	118.4 ± 36.3	118.1 ± 33.6	0.83
Triglycerides (mg/dL)	187.0 ± 134.1	113.5 ± 66.6	<0.001

NFS (NAFLD fibrosis score, NFS = -1.675 + 0.037 x age(year) + 0.094 x BMI (kg/m^2^) + 1.13 x impaired fasting glycemia or diabetes (yes = 1, no = 0) + 0.99 x AST/ALT ratio—0.013 x PLT (10^9^/L) - 0.66 x ALB (g/dL); high probability (>0.676), indeterminate (0.676- -1.455), low probability (< -1.455) of advanced fibrosis

Demographic characteristics of subjects with NAFLD based on risk of fibrosis are summarized in Table A in S1 File, while clinical and laboratory characteristics are summarized in Table B in S1 File. NAFLD subjects with advanced fibrosis were more likely to be male, older, non-Hispanic White or Black, born in the U.S., have lower income, have previous smoking exposure, and have a higher education level (Table A in S1 File). Subjects with advanced fibrosis were also more likely to have hypertension, metabolic syndrome, diabetes whether controlled or uncontrolled, kidney failure, arthritis, ischemic heart disease, congestive heart failure, stroke, COPD, and cancer. In addition, subjects with advanced fibrosis were more likely to have higher BMI, waist circumference, total bilirubin, creatinine, HbA1c, fasting glucose, fasting insulin, HOMA-IR, and HDL cholesterol (Table B in S1 File).

### Prevalence of NAFLD and risk factors for advanced fibrosis in subjects with NAFLD

Among 6,000 continuous NHANES participants aged 18 years or older, the prevalence of NAFLD was 30.0% using a USFLI cut-off of ≥30. Of the subjects with NAFLD, 44.0% and 10.3% had an NFS consistent with a low and high probability of advanced fibrosis, respectively (Table 2). Univariate and multivariate analysis of predictors of advanced fibrosis (NFS>0.676) among patients with NAFLD are shown in Table 3. The independent risk factors for advanced fibrosis in subjects with NAFLD are male sex, as well as lower education level. Interestingly, Mexican American ethnicity was a negative independent predictor for advanced fibrosis.

**Table 3:** Predictors for advanced fibrosis in participants with NAFLD in the U.S. National Health and Nutrition Examination Survey, 1999–2012 (n = 1936).

	Univariate analysis		Multivariate analysis	
	OR (95% CI)	*p*-value	OR (95% CI)	*p*-value
**Male**	0.70 (0.50–0.99)	0.045	0.70 (0.49–0.99)	0.046
**Ethnicity**				
Non-Hispanic White (Referent)				
Non-Hispanic Black	1.25 (0.78–2.01)	0.35	1.19(0.73–1.93)	0.478
Mexican American	0.53 (0.35–0.81)	0.003	0.45(0.30–0.68)	<0.001
**Education level**				
>High school (Referent)				
≤High school	1.51 (1.08–2.13)	0.018	1.60 (1.12–2.31)	0.011
**Smoking status**				
Never (Referent)				
Current/former	1.24 (0.89–1.72)	0.21	1.24(0.90–1.71)	0.189

### Mortality in NAFLD subjects and by risks for advanced fibrosis

All-cause mortality at 5 and 8 years for subjects with NAFLD were significantly higher than corresponding mortality for those without NAFLD. Similar trends were found for cardiovascular-related and cancer-related 5 and 8-year mortality in subjects with NAFLD compared to those without NAFLD (Table C in S1 File).

**Fig 1:**
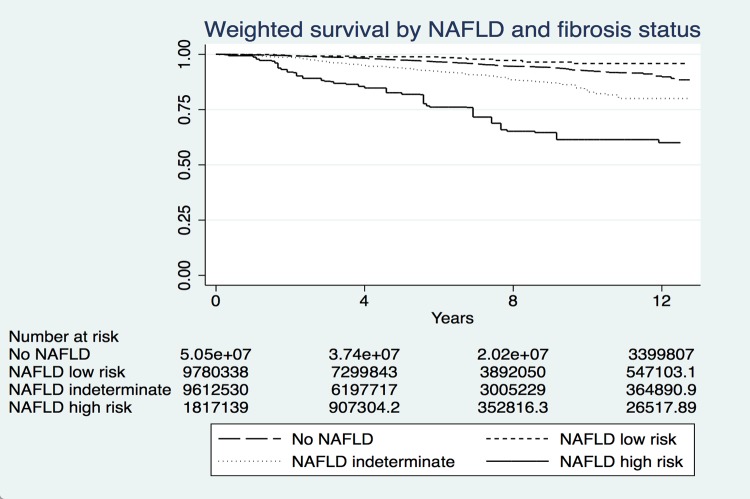
Weighted survival by NAFLD and fibrosis status based on NFS cutoffs in the National Health and Nutrition Examination Survey, 1999–2010. NFS cutoffs for advanced fibrosis: low risk <-1.455, indeterminate risk -1.455–0.676, high risk >0.676.No NAFLD vs. NAFLD low risk: p = 0.015| No NAFLD vs. NAFLD indeterminate risk: p<0.001. No NAFLD vs. NAFLD high risk: p<0.001 |NAFLD low risk of advanced fibrosis vs NAFLD high risk of advanced fibrosis: p<0.001|

There were also significant differences of all-cause mortality rates among patients by presence of NAFLD and by degree of fibrosis among those with NAFLD weighted analysis for the general U.S. population (Fig 1). The 5 and 8-year all-cause mortality for subjects with NAFLD and high risk for advanced fibrosis were significantly higher than those of subjects without NAFLD, who interestingly had higher mortality rate than subjects with NAFLD but low risk for fibrosis, since the low fibrosis NAFLD subjects tended to be significantly younger (43.7 vs 47.3, p<0.0001). Likewise, 5-year and 8-year cardiovascular-related and cancer-related mortality for NAFLD subjects with high risk for advanced fibrosis were significantly higher than those with NAFLD and low risk for fibrosis (Table D in S1 File).

Of the variables of interest, sex, education level, smoking, NAFLD with advanced fibrosis, cardiovascular disease, cancer, and chronic obstructive pulmonary disease fit the Cox proportionality assumption, as the Cox predicted survival curves matched very closely to the Kaplan-Meier survival curves. Univariate and multivariate analysis of predictors of mortality are shown in Table 4. In multivariate model that is also inclusive of sex, ethnicity, education level, smoking status, cardiovascular disease, cancer, and COPD, high risk for advanced fibrosis was a significant independent predictor of increased all-cause mortality, with high-risk patients having over three folds higher likelihood of dying than those without NAFLD (HR = 3.13, 95% CI 1.93–5.08, *p*<0.001) but not NAFLD subjects with only low risk of fibrosis. Less than high school education and current or past history of smoking were also significantly associated with higher mortality.

**Table 4:** Risk factors for overall mortality in the U.S. National Health and Nutrition Examination Survey, 1999–2010 (n = 5,086).

	Univariate analysis		Multivariate analysis	
Variable	HR (95% CI)	*p*-value	HR (95% CI)	*p-*value
**Male**	1.25 (0.99–1.58)	0.057	1.13 (0.85–1.50)	0.405
**Education level**				
>High school (Referent)				
≤High school	1.94 (1.54–2.46)	<0.001	1.71 (1.30–2.25)	<0.001
**Smoking status**				
Never (Referent)				
Current/former	1.91 (1.51–2.40)	<0.001	1.37 (1.03–1.82)	0.028
**NAFLD status**				
No NAFLD (Referent)				
Low risk of advanced fibrosis	0.48 (0.26–0.88)	0.015	0.41 (0.22–0.76)	0.004
High risk of advanced fibrosis	7.30 (4.81–11.1)	<0.001	3.13 (1.93–5.08)	<0.001
**Cardiovascular Disease**	5.25 (4.00–6.88)	<0.001	3.89 (2.69–5.63)	<0.001
**Cancer**	3.62 (2.77–4.74)	<0.001	2.64 (1.87–3.72)	<0.001
**Chronic Obstructive Pulmonary Disease**	3.06 (2.20–4.27)	<0.001	1.66 (1.07–2.56)	0.023

## Discussion

Our study examined the prevalence of NAFLD, advanced fibrosis and mortality using the recent NHANES 1999–2012 cycles. This is also the first population-based study using previously validated noninvasive marker panels, USFLI and NFS, to identify risk factors for advanced fibrosis in subjects with NAFLD. In this multiethnic, national, U.S. population-based study, we found the prevalence of NAFLD to be 30.0%, comparable to results from investigations relying on imaging studies[8,33]. In subjects with NAFLD, male sex and Mexican American ethnicity were shown to be protective factors against advanced fibrosis. On the other hand, a lower education level was shown to be a significant risk factor for advanced fibrosis in those with NAFLD. We also found that subjects with NAFLD and a high risk of advanced fibrosis have a higher risk of mortality compared to patients without NAFLD but not NAFLD subjects with low risk for advanced fibrosis.

As shown by prior studies as well as this study, Mexican American ethnicity is associated with higher risk for NAFLD but not necessarily higher risk for advanced fibrosis among those with NAFLD and that the majority of Mexican American subjects with hepatic steatosis tend to have normal ALT levels [33–36]. Similar ethnic effect has also been shown for African Americans with chronic hepatitis C[37]. Genetic studies should be considered to elucidate the mechanism for these findings.

In regards to mortality, there have been conflicting findings on the impacts of NAFLD on mortality using the NHANES III database, depending on how NAFLD is defined[33,38–40]. One study utilized the NHANES III data (1988–1994) and defined NAFLD as subjects with elevated serum aminotransferases in the absence of alcohol abuse, elevated transferrin saturation, and positivity for viral hepatitis[38]. This study found that NAFLD was associated with higher overall and liver-related mortality than the general U.S. population[38]. However, two recent studies that utilized the same NHANES III dataset with follow-up of up to 23 years have found different results when NAFLD was defined based on ultrasound findings of hepatic steatosis[33,39]. Both studies found NAFLD per se was not associated with increased mortality compared to the general population, but rather the presence of significant fibrosis (using non-invasive markers such as NFS, FIB-4 or APRI) were significant predictors of higher mortality[33,39] but mostly from cardiovascular diseases. Another more recent study using the NHANES III database showed that severe hepatic steatosis on ultrasound and elevated liver enzymes were associated with increased mortality from liver diseases, but not with all-cause, cardiovascular related, cancer related, or diabetes related mortality[40]. Our study is the first study to examine mortality in patients with NAFLD using more recent data than the NHANES III dataset. Our study utilized the most recent NHANES datasets from 1999–2010. Compared to the previous studies using the NHANES III dataset, our mortality findings using the NFS to identify NAFLD subjects with low and high risk for advanced fibrosis show similar trends. This is also the first study that applied this fibrosis scoring system in a population-based cohort of NAFLD identified via the USFLI, which has been previously validated to correlate well with presence of NAFLD in the United States population. This index does not rely on imaging studies which may not be readily available in many clinical settings. Another population-based study in Olmsted County found increased mortality and liver-related death in patients with NAFLD compared to the general population, with malignancy and ischemic heart disease as the main cause of death in both groups[41]. This study defined NAFLD as fatty infiltration of the liver confirmed by imaging/liver biopsy or patients diagnosed with cryptogenic cirrhosis who also had metabolic syndrome and used liver biopsy to identify patients with advanced fibrosis. In our study, we also found NAFLD to associate with increased risk of cardiovascular and cancer-related mortality. In addition, we found patients with NAFLD that are at low risk of fibrosis had lower mortality than subjects without NAFLD. Subjects with NAFLD and low risk of fibrosis tended to be significantly younger. As such, the NAFLD with low risk of fibrosis group had a lower cardiovascular mortality compared to the no NAFLD group, which may explain the lower risk of all-cause mortality compared to the no NAFLD group.

Our study has several limitations. Because it is difficult to perform liver biopsy in a large population-based study sample, we used non-invasive measures such as NFS to identify advanced fibrosis as opposed to the gold standard of liver histology. Similarly, since abdominal imaging is not always available in population-based study, we use USFLI to identify subjects with NAFLD. A large portion of the patient population was omitted due to missing data for required for these noninvasive assessments. Another limitation of the study is the use of the 2011 Public-Use Linked Mortality Files rather than the 2011 Restricted-Use Linked Mortality Files. These public-use files suffer from a lack of information in identifying additional causes of death, including liver related deaths and the files have undergone data perturbation to help protect the identities of those who participated in NHANES. However, these techniques did not have a very large effect on the data, with both the 2011 Restricted-Use Linked Mortality Files and the 2011 Public-Use Linked Mortality Files showing similar percentages of deaths by various causes[24]. Since the mortality data was last released in December 2011, the availability of more recent data is limited. Of the six survey cycles with mortality data, only four cycles have at least 5 years follow up, which is the drawback of using such a recent dataset.

In conclusion, our study utilized data from a more recent NHANES dataset from 1999–2012, utilized the USFLI to estimate NAFLD prevalence and showed that the prevalence of NAFLD appears to remain stable at 30%. We also found that only a minority (10%) of NAFLD subjects were at risk for advanced fibrosis, and only these subjects would be at increased risk for all-cause mortality as well as cardiovascular and cancer related mortality. Mexican American ethnicity was at higher risk for NAFLD, but those with NAFLD actually had lower risk for advanced fibrosis compared to non-Hispanic Whites. Patients with NAFLD and risk for advanced fibrosis should be especially targeted for life-style and other available medical intervention.

## Supporting information

S1 File Table A: Demographic characteristics of participants with NAFLD by fibrosis status based on NFS cutoffs in the U.S. National Health and Nutrition Examination Survey, 1999–2012. Table B: Lab and clinical characteristics of participants by NAFLD and Fibrosis status based on NFS cutoffs in the U.S. National Health and Nutrition Examination Survey, 1999–2012. Table C: Mortality in subjects by NAFLD status in the National Health and Nutrition Examination Survey 1999–2010. Table D: Mortality by Fibrosis Status in subjects with NAFLD in the National Health and Nutrition Examination Survey 1999–2010.
(DOCX)
