# Supplementary material for: Prevalence of non-alcoholic fatty liver disease and risk factors for advanced fibrosis and mortality in the United States
Source: PLoS One. 2017 Mar 27;12(3):e0173499. doi: 10.1371/journal.pone.0173499 (PMC5367688; doi:10.1371/journal.pone.0173499)
Supplement: S1 File — Table A: Demographic characteristics of participants with NAFLD by fibrosis status based on NFS cutoffs in the U.S. National Health and Nutrition Examination Survey, 1999–2012. Table B: Lab and clinical characteristics of participants by NAFLD and Fibrosis status based on NFS cutoffs in the U.S. National Health and Nutrition Examination Survey, 1999–2012. Table C: Mortality in subjects by NAFLD status in the National Health and Nutrition Examination Survey 1999–2010. Table D: Mortality by Fibrosis Status in subjects with NAFLD in the National Health and Nutrition Examination Survey 1999–2010. (DOCX) [file pone.0173499.s001.docx]

**Supplementary Information**

**Table A: Demographic characteristics of participants with NAFLD by fibrosis status based on NFS cutoffs in the U.S. National Health and Nutrition Examination Survey, 1999-2012**

| Variable | NAFLD low risk of advanced fibrosis (n=739) | NAFLD indeterminate risk of advanced fibrosis (n=919) | NAFLD high risk of advanced fibrosis (n=245) | *p-*value^1^ | *p-*value^2^ | *p-value^3^* |
| --- | --- | --- | --- | --- | --- | --- |
| Age (years) | 43.7 ± 13.2 | 58.8 ±14.2 | 67.9 ± 13.4 | <0.001 | <0.001 | <0.001 |
| Male | 61.8 | 58.7 | 51.6 | 0.282 | 0.015 | 0.140 |
| Ethnicity | | | | <0.001 | <0.001 | 0.071 |
| Non-Hispanic White | 72.1 | 81.3 | 83.8 |  |  |  |
| Non-Hispanic Black | 5.6 | 5.5 | 7.5 |  |  |  |
| Mexican American | 13.1 | 6.1 | 5.6 |  |  |  |
| Other Hispanic | 4.8 | 3.8 | 2.3 |  |  |  |
| Other race | 4.6 | 3.4 | 0.8 |  |  |  |
| Foreign born | 17.1 | 10.9 | 6.4 | <0.001 | <0.001 | 0.023 |
| United States citizen | 89.8 | 95.2 | 97.5 | <0.001 | <0.001 | 0.041 |
| Served in the U.S. military | 11.2 | 24.1 | 28.4 | <0.001 | <0.001 | <0.001 |
| Income | | | | 0.189 | <0.001 | 0.015 |
| < $45,000 | 38.9 | 44.9 | 60.0 |  |  |  |
| $45,000-74,999 | 26.6 | 22.9 | 20.2 |  |  |  |
| ≥ $75,000 | 34.5 | 32.2 | 19.8 |  |  |  |
| Poverty income ratio < 1.0 | 12.8 | 8.5 | 9.0 | 0.460 | 0.170 | 0.828 |
| Marriage status | | | | 0.011 | <0.001 | 0.140 |
| Legally married | 67.3 | 70.4 | 58.6 |  |  |  |
| Divorced/separated | 9.2 | 8.4 | 14.4 |  |  |  |
| Never married | 14.6 | 8.3 | 3.8 |  |  |  |
| Other | 8.9 | 13.0 | 23.2 |  |  |  |
| Smoking exposure | | | | 0.005 | 0.031 | 0.848 |
| Never | 63.8 | 54.9 | 54.1 |  |  |  |
| Current/former | 36.2 | 45.1 | 45.9 |  |  |  |
| Education level | | | | 0.981 | 0.029 | 0.023 |
| ≤ High school | 59.5 | 59.4 | 49.2 |  |  |  |
| > High school degree | 40.5 | 40.6 | 50.8 |  |  |  |

NFS (NAFLD fibrosis score, NFS = -1.675 + 0.037 x age(year) + 0.094 x BMI (kg/m^2^) + 1.13 x impaired fasting glycemia or diabetes (yes=1, no=0) + 0.99 x AST/ALT ratio - 0.013 x PLT (10^9^/L) - 0.66 x ALB (g/dL); high risk of stage 3-4 (>0.676), indeterminate (0.676- -1.455), low risk of stage 3-4 (< -1.455)

^1^NAFLD low risk of fibrosis vs. NAFLD indeterminate risk of fibrosis

^2^NAFLD low risk of fibrosis vs. NAFLD high risk of fibrosis

^3^NAFLD indeterminate risk of fibrosis vs. NAFLD high risk of fibrosis

**Table B: Lab and clinical characteristics of participants by NAFLD and Fibrosis status based on NFS cutoffs in the U.S. National Health and Nutrition Examination Survey, 1999-2012**

| Variable | NAFLD low risk of advanced fibrosis (n=739) | NAFLD indeterminate risk of advanced fibrosis (n=919) | NAFLD high risk of advanced fibrosis (n=245) | *p-*value^1^ | *p-*value^2^ | *p-*value^3^ |
| --- | --- | --- | --- | --- | --- | --- |
| Hypertension | 15.6 | 24.6 | 26.9 | <0.001 | 0.003 | 0.584 |
| Hypercholesterolemia | 46.4 | 31.2 | 15.1 | <0.001 | <0.001 | <0.001 |
| Hyperlipidemia | 53.7 | 49.8 | 41.7 | 0.213 | 0.025 | 0.074 |
| Metabolic syndrome | 44.4 | 65.1 | 66.4 | <0.001 | <0.001 | 0.776 |
| Diabetes | 6.1 | 32.0 | 50.9 | <0.001 | <0.001 | <0.001 |
| Controlled (HbA1c<6.5%) | 2.5 | 13.4 | 23.2 | <0.001 | <0.001 | <0.001 |
| Uncontrolled (HbA1c≥6.5%) | 3.6 | 18.6 | 27.8 |  |  |  |
| Kidney failure | 0.8 | 2.5 | 5.0 | 0.011 | <0.001 | 0.060 |
| Asthma | 14.4 | 15.8 | 15.6 | 0.49 | 0.72 | 0.955 |
| Arthritis | 23.4 | 46.2 | 53.3 | <0.001 | <0.001 | 0.095 |
| Ischemic heart disease | 5.8 | 13.2 | 28.4 | <0.001 | <0.001 | <0.001 |
| Congestive heart failure | 1.9 | 4.9 | 11.6 | 0.010 | <0.001 | <0.001 |
| Stroke | 2.5 | 4.1 | 7.4 | 0.122 | 0.002 | 0.047 |
| Chronic obstructive pulmonary disease | 5.9 | 9.6 | 17.7 | 0.044 | <0.001 | 0.003 |
| Cancer | 5.7 | 15/3 | 26.8 | <0.001 | <0.001 | 0.002 |
| BMI (kg/m^2^) | 32.3 ± 5.4 | 34.4 ± 6.7 | 37.8 ± 10.0 | <0.001 | <0.001 | <0.001 |
| Waist circumference (cm) | 108.3 ± 12.0 | 114.3 ± 11.9 | 121.8 ± 19.0 | <0.001 | <0.001 | <0.001 |
| Albumin (g/dL) | 4.3 ± 0.3 | 4.2 ± 0.3 | 4.1 ± 0.3 | <0.001 | <0.001 | <0.001 |
| Alanine aminotransferase (U/L) | 35.8 ± 22.5 | 29.0 ± 14.9 | 25.4 ± 16.3 | <0.001 | <0.001 | 0.008 |
| Aspartate aminotransferase (U/L) | 27.5 ± 13.0 | 26.3 ± 11.3 | 27.4 ± 14.2 | 0.105 | 0.892 | 0.332 |
| Alkaline phosphatase (U/L) | 74.4 ± 19.7 | 73.1 ± 22.7 | 70.9 ± 28.1 | 0.302 | 0.057 | 0.243 |
| Gamma-glutamyl transferase (U/L) | 40.6 ± 31.9 | 35.6 ± 38.9 | 41.0 ± 59.5 | 0.013 | 0.907 | 0.108 |
| Platelet (10^9^/L) | 292.9 ± 62.0 | 240.7 ± 53.8 | 197.9 ± 56.9 | <0.001 | <0.001 | <0.001 |
| Total bilirubin (mg/dL) | 0.74 ± 0.29 | 0.78 ± 0.31 | 0.80 ± 0.34 | 0.031 | 0.014 | 0.393 |
| Creatinine (mg/dL) | 0.87 ± 0.19 | 0.94 ± 0.36 | 1.05 ± 0.42 | <0.001 | <0.001 | 0.001 |
| HbA1c | 5.6 ± 0.7 | 6.1 ± 1.3 | 6.4 ± 1.4 | <0.001 | <0.001 | 0.007 |
| Fasting glucose (mg/dL) | 104.0 ± 21.2 | 123.6 ± 42.1 | 130.7 ± 40.2 | <0.001 | <0.001 | 0.024 |
| Fasting insulin (uU/mL) | 20.1 ± 9.6 | 20.6 ± 12.5 | 26.3 ± 43.4 | 0.540 | <0.001 | 0.049 |
| HOMA-IR | 5.2 ± 2.8 | 6.3 ± 4.7 | 8.6 ± 14.8 | <0.001 | 0.001 | 0.013 |
| Total cholesterol (mg/dL) | 208.8 ± 42.4 | 195.9 ± 42.3 | 179.6 ± 40.3 | <0.001 | <0.001 | <0.001 |
| HDL cholesterol (mg/dL) | 44.4 ± 10.8 | 45.6 ± 12.0 | 47.7 ± 13.3 | 0.060 | 0.003 | 0.034 |
| LDL cholesterol (mg/dL) | 126.8 ± 33.0 | 114.9 ± 35.7 | 101.2 ± 35.1 | <0.001 | <0.001 | <0.001 |
| Triglycerides (mg/dL) | 199.3 ± 145.2 | 180.4 ± 117.0 | 163.1 ± 112.1 | 0.039 | 0.001 | 0.027 |

NFS (NAFLD fibrosis score, NFS = -1.675 + 0.037 x age(year) + 0.094 x BMI (kg/m^2^) + 1.13 x impaired fasting glycemia or diabetes (yes=1, no=0) + 0.99 x AST/ALT ratio - 0.013 x PLT (10^9^/L) - 0.66 x ALB (g/dL); high risk of stage 3-4 (>0.676), indeterminate (0.676- -1.455), low risk of stage 3-4 (< -1.455)

^1^NAFLD low risk vs. NAFLD indeterminate risk of advanced fibrosis

^2^NAFLD low risk vs. NAFLD high risk of advanced fibrosis

^3^NAFLD indeterminate risk vs. NAFLD high risk of advanced fibrosis

**Table C: Mortality in subjects by NAFLD status in the National Health and Nutrition Examination Survey 1999-2010**

| Variable | NAFLD | No NAFLD | *p-*value |
| --- | --- | --- | --- |
| Months of follow up | 75.4 ± 42.7 | 80.7 ± 40.4 | 0.001 |
| **All-cause mortality (%)** | | | <0.001 |
| 5 year | 4.9 | 2.6 |  |
| 8 year | 9.4 | 5.5 |  |
| **Cardiovascular mortality (%)** | | | 0.049 |
| 5 year | 0.9 | 0.6 |  |
| 8 year | 1.5 | 1.0 |  |
| **Cancer mortality (%)** | | | 0.001 |
| 5 year | 1.5 | 0.6 |  |
| 8 year | 2.8 | 1.1 |  |

**Table D: Mortality by Fibrosis Status in subjects with NAFLD in the National Health and Nutrition Examination Survey 1999-2010**

| Variable | No NAFLD | NAFLD low risk advanced fibrosis | NAFLD indeterminate risk advanced fibrosis | NAFLD high risk advanced fibrosis | *p-*value^1^ | *p*-value^2^ | *p-* value^3^ | *p-* value^4^ |
| --- | --- | --- | --- | --- | --- | --- | --- | --- |
| Months follow up | 80.7 ± 40.4 | 82.5 ± 39.1 | 71.6 ± 44.0 | 57.6 ± 44.0 | 0.390 | <0.001 | <0.001 | <0.001 |
| **All cause mortality (%)** | | | | | 0.015 | <0.001 | <0.001 | <0.001 |
| 5 year | 2.6 | 1.1 | 6.1 | 18.1 |  | | | |
| 8 year | 5.5 | 2.8 | 11.6 | 35.0 |  |  |  |  |
| **Cardiovascular mortality (%)** | | | | | 0.001 | 0.003 | <0.001 | <0.001 |
| 5 year | 0.6 | <0.1 | 1.0 | 5.1 |  | | | |
| 8 year | 1.0 | <0.1 | 2.0 | 6.2 |  |  |  |  |
| **Cancer mortality (%)** | | | | | 0.700 | <0.001 | <0.001 | 0.009 |
| 5 year | 0.6 | 0.7 | 2.0 | 3.5 |  | | | |
| 8 year | 1.1 | 1.4 | 3.8 | 5.9 |  |  |  |  |

^1^ No NAFLD vs low risk of advanced fibrosis

^2^No NAFLD vs indeterminate risk of advanced fibrosis

^3^ No NAFLD vs high risk

^4^ Low risk vs high risk of advanced fibrosis
